# Surgical Fixation of Clavicle Shaft Fractures Using Superior Locking Plates With Lateral End Extension: A Retrospective Study

**DOI:** 10.7759/cureus.30054

**Published:** 2022-10-08

**Authors:** Emmanuel Ago, Vetri Thiruvasagam, Nasir Shah, Ravi Badge

**Affiliations:** 1 Trauma and Orthopaedics, Warrington and Halton Hospitals National Health Service (NHS) Foundation Trust, Warrington, GBR

**Keywords:** surgical fixation devices, superior locking plate, surgical managment, clavicle surgery, clavicle fracture

## Abstract

Background

Reports of high rates of non-union with poor functional outcomes following non-operative management of clavicle fractures have resulted in a shift of opinion towards the promising outcomes of surgical fixation. Varied implant choices with varying reports of success and associated complications have resulted in no definitive consensus on the choice of the ideal implant.

Materials and Methods

This is a retrospective study of clavicle shaft fractures stabilized using a Superior Clavicle Locking plate with lateral extension in 40 active adult patients, predominantly male, with an average age of 36.7 years.

Results

Post-operatively, early mobilization was initiated and on final discharge, there were no complaints of pain. All patients returned to their pre-injury levels of activity by four months, with all having achieved, essentially, a full range of movement by eight weeks post-operatively. Radiological union was observed in all patients by 5 months, except one. The QuickDASH scores of all the patients were less than 25 on discharge.

Conclusion

When surgical stabilization is considered in the management of active adults with clavicle shaft fractures, the superior clavicle locking plate with lateral extension appears to be a suitable implant by providing stable fixation lateral to the fracture, which is difficult with a regular locking plate.

## Introduction

The optimal management of clavicle fractures has been the subject of debate for decades with a recent shift in preference toward surgical stabilization over conservative management [1]. With clavicle fractures accounting for 4%-10% of all fractures, there has been a plethora of published evidence in support of both surgical and conservative management, however, there remains no definitive consensus on a single best approach [2].

The clavicle is S-shaped, and forward-facing is convex medially and concave laterally. The cross-section is tubular in the middle and flat towards its lateral end. Anatomical variations have been observed based on age, sex, and race, in the morphology of the clavicle and its medullary canal [3,4]. The use of a single implant design to accommodate these anatomical variations has been not successful so far, especially with respect to plating [5]. The inadequacy of the implant leads to complications including soft tissue irritation, non-union, implant loosening, and deep-seated infection [6].

Due to the unique morphology of the clavicle bone, which makes conventional plates and screws unsuitable, there has been an evolution in plate design with more recent models including locking plates with a lateral extension that allow the purchase of multiple screws at the lateral end of the clavicle providing a stable fixation of the fracture. This study aims to report our experience, results, and outcomes following Open Reduction and Internal Fixation of Diaphyseal clavicle fractures using a Superior Clavicle Locking Compression Plate (LCP) with lateral extension.

## Materials and methods

**Figure 1:**
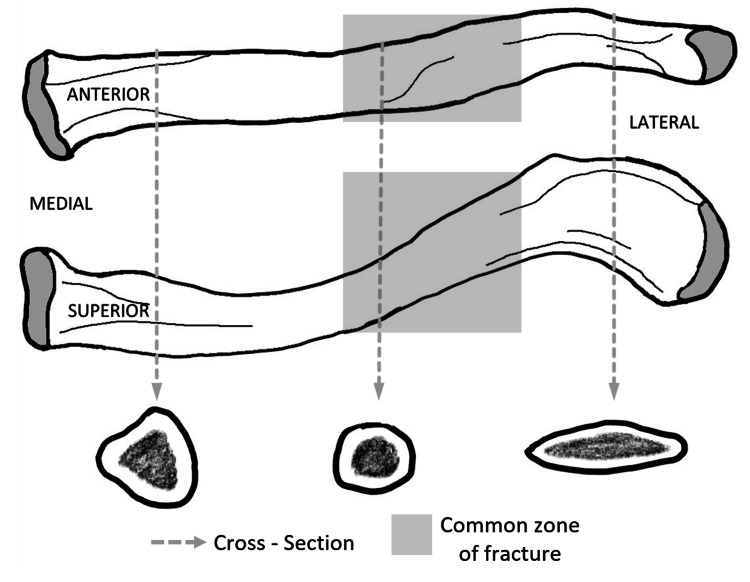
Anatomical morphology of the clavicle.

A retrospective observational study of surgical fixation for diaphyseal clavicle fractures in a single institution over two years between October 2016 to October 2018 using the SynthesTM 3.5/2.7mm Superior Clavicle LCP with lateral extension was carried out. Following a presentation to the Emergency Department, patients with clavicle fractures associated with open wounds, skin tenting, or neurovascular injury, were admitted for further management. All others were followed up within a week in the fracture clinic. At the clinic, patients who led active lifestyles presenting with displaced, shortened, or comminuted diaphyseal clavicle fracture were offered treatment options, including conservative and surgical with their associated risks and benefits. This study was approved by the local clinical audit department. Ethical approval was not required, as data was collected for quality improvement and audit purposes; there was no direct involvement of human subjects. An anatomical morphology of the clavicle is shown in Figure 1.

Implant used

The SynthesTM 3.5/2.7mm Superior Clavicle LCP with Lateral extension are anatomically contoured plates with designs for the right and left sides. The plates are available in variable lengths and have between three to eight combi-holes to accommodate 3.5mm locking and working cortical screws. The ‘lateral extension’ is designed to sit on the lateral end of the clavicle with three pairs of screw holes, each in different orientations, to accommodate 2.7mm locking screws or cortical screws. The medial and lateral third of the plate has reconstruction segments that can be used to bend the plate if required, usually to reduce plate prominence. Other implant manufacturers have similar designs with lateral end extensions to accommodate screws in the lateral end of the clavicle.

Surgical technique

In all the cases, the procedure was carried out under general anesthesia in the beach chair position, with the fracture side and position confirmed using an Image Intensifier. The surgical site was infiltrated with local anesthetic and adrenaline. A curvilinear incision centered over the clavicle fracture was used and extended to the lateral end of the clavicle. The platysma was incised along the line of the incision and tagged with sutures. Supraclavicular nerves were identified, and all possible measures are taken to protect them. The fracture was exposed by elevating the clavipectoral fascia minimally and the superior surface cleared with a periosteal elevator. 

None of the fragments were detached from their soft-tissue attachments. Butterfly fragments amenable to lag screw fixation were stabilized using a 2.7mm or 3.5mm cortical screw. Alternatively, when the use of a lag screw proved unfeasible, a fiber wire is passed and tied around the butterfly fragment and end of the fracture, held temporarily using a clamp or a K-wire. The fracture was reduced and stabilized with a Synthes 3.5/2.7mm Superior Clavicle LCP with Lateral extension in neutral mode, using both locking and cortical working screws. Three 3.5mm screws were used medial to the fracture site. Lateral to the fracture site one or more 3.5mm screws were used along with at least five 2.7mm locking screws in the lateral extension part of the plate.

Postoperative regime

After wound closure and pressure dressing, the arm was placed in a broad arm sling. Patients were commenced on pendulum exercises before discharge. Visual Analog Scores (VAS) on discharge were taken from the patients’ notes. Active shoulder movements within pain limits were permitted for the first three weeks. The arm sling was kept for three weeks, primarily for comfort, following which active unrestricted mobilization of the shoulder was permitted. Non-weight bearing, particularly avoidance of heavy lifting and contact sports, was advised till review at six weeks when there was evidence of bone healing on check radiographs. Patients were reviewed at 2, 6, 12, and 24 weeks, with radiographs from six weeks on each visit. Patients were followed up in the clinic for six months following the procedure, at which point fracture union was observed radiologically in all patients. On discharge from care, a QuickDASH score was used to assess their shoulder function. They were subsequently reviewed in the physiotherapy clinic until they achieved maximum shoulder range of movements.

Statistics

No statistical analysis was performed.

## Results

Trauma mechanism 

Forty patients, 33 males, and seven females, who met the inclusion criteria; closed injuries, neurovascularly intact and without significant skin tenting, underwent clavicle stabilization using SynthesTM 3.5/2.7mm Superior Clavicle Plate with lateral extension in the abovementioned period. The average age of patients was 36.7 years, ranging between 17 to 73 years, with the mechanism of trauma presented in Table 1.

**Table 1:** Trauma mechanism for patients presenting with clavicle fractures within out cohort.

Mechanism	Prevalence
Cycling	30 (75%)
Sport related (rugby, football, etc.)	4 (10%)
Fall from standing height	4 (10%)
Motorbike accident	2 (5%)

Fracture classification

**Figure 2:**
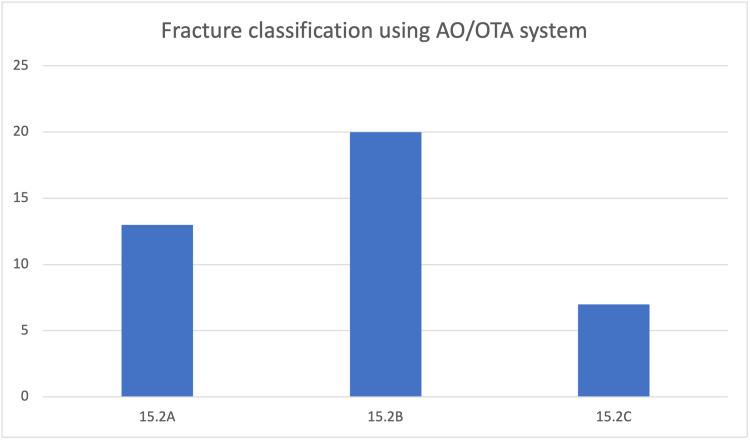
Classification of fracture patterns observed using the AO/OTA classification system.

Fractures were classified using the AO/OTA classification system (Figure 2) [7]. The majority of Type 15.2B fractures, had the butterfly fragments displaced and oriented vertically at almost 90 degrees, like a kickstand. Type 15.2A fractures were more predominant after the fourth decade of life and required surgical intervention for gross shortening in two cases and displacement greater than 2cm in the remainder of the cases. The majority of the fractures were oriented towards the middle/distal third junction of the clavicle and were usually not amenable to lag screw fixation. 

Postoperative phase

In the postoperative period, the VAS dropped to zero or one and was consistently low at the two-week review for all the patients. There were no iatrogenic injuries. None of the patients had any pain at six weeks, except one with a VAS of one. None of the patients had any pain at the final discharge. 

**Figure 3:**
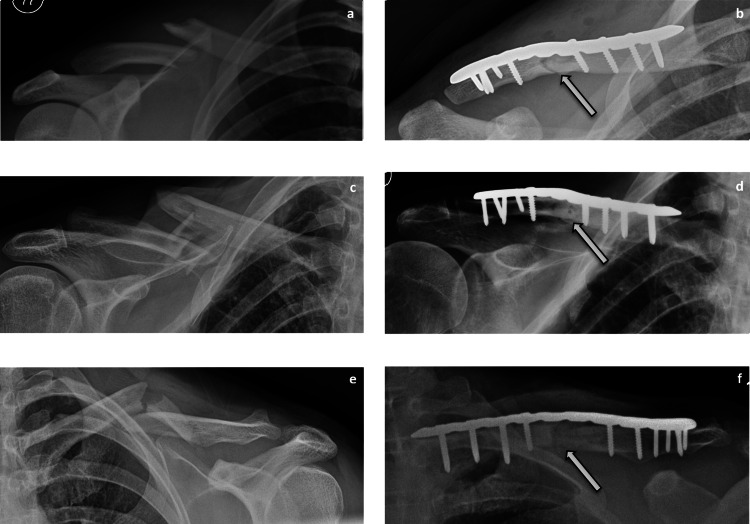
Pre- and post-clavicle fixation radiographs demonstrating union, as denoted by arrows.

All 40 patients were followed up for six months postoperatively, however open access to follow-up in the clinic was provided in the event of patient concern. At six weeks, 33 patients demonstrated a virtually full range of movements in the shoulder. At 12 weeks, 38 patients had regained full range of motion and by four months were undertaking pre-injury levels of activity. Two patients (both above 60 years of age) had terminal restrictions of abduction and forward flexion by 30 degrees. Radiological union was achieved in 37 patients by 4 months (Figure 3). Delayed union at 20 weeks was observed in two patients. Clinically, all 40 patients had no pain at the fracture site by 12 weeks. 

Complications

We observed no major complications such as wound infection, shoulder instability, or damage to soft tissues, including rotator cuff muscles. We observed one instance of the following complications respectively; non-union, soft tissue irritation, and implant failure secondary to repeated trauma.

One patient, who represented following final follow-up at six months, wanted implant removal at 10 months for soft tissue irritation. Another, a 45-year-old active male, had a fall and sustained a broken plate at eight months, requiring revision surgery using a similar implant with bone grafting. Following the revision, the patient had no further pain and the fracture healed with the patient returning to his routine activities at four months. 

Though the supraclavicular nerves were protected intra-operatively, all patients complained of varying levels of numbness over the infraclavicular region. However, at the final follow-up, the numbness had resolved or settled to barely noticeable levels, in all patients. 

Patient-reported outcomes

Patient-reported outcomes were measured using QuickDASH scores, which were requested on the final review at six months postoperatively. Twenty-seven of the 40 participants responded, with all scores being 25 or less; 0 in 23 patients and 25 in four patients. 

## Discussion

A systematic review by Virtanen et al. of literature published between 1966 and March 2011, demonstrated only slightly better outcomes in surgically fixed clavicle fractures and this benefit appeared to diminish after six months postoperatively. However, higher rates of fracture union were achieved with fixation, with evidence supporting fixation in young and active patients as it facilitated the return to previous activity levels in the shortest possible time [8]. There is good evidence for the conservative management of clavicle fractures, with immobilization for 4 to 6 weeks, however, it is increasingly being limited to undisplaced diaphyseal fracture patterns due to increased reports of patient dissatisfaction and high rates of non-union, 5.9% in undisplaced fractures and 18% in displaced fractures [8].

Operative stabilization has become the mainstay for the management of displaced or comminuted clavicle fractures due to earlier return to work, improved patient satisfaction, and improved outcomes [9]. Studies considering both intramedullary and plate fixation provide inconclusive reports, asserting both techniques to have equivocal outcomes [10].

Drosdowech et al., in their biomechanical study, established superior clavicle locking plates to provide the most rigidity and resistance to bending compared to other plate constructs, and significantly more than intramedullary fixations, especially in the presence of comminution [11]. Plate fixation has been singled out for its high infection rate, non-union and wound healing problems, and re-operation rates, as high as 18.8% [12]. Associated complications of plate fixation are attributed to the stripping of soft tissues while plating, and hardware prominence, as well as the common occurrence of implant removal to address adverse effects [6]. 

Our experience, however, shows that stabilization of clavicle fractures with the SynthesTM 3.5/2.7mm Superior Clavicle LCP with Lateral extension has been excellent in terms of radiological and patient outcomes. As alluded to in our results, we had a single case of non-union (2.5%) within the entire cohort, which was asymptomatic, and one patient require implant removal (2.5%) for soft tissue irritation at 10 months following fixation, with radiological evidence of fracture union. We observed no wound infections or significant neurovascular deficit. Early mobilization of the shoulder joint was possible due to the stable construct of the fixation using the plate with lateral extension.

Although 80% of clavicle fractures involve the middle third, a biomechanical study has demonstrated clavicle fractures to most commonly occur at the middle/distal third junction of the bone with the superior-anterior aspect of the shaft as the tension site [13]. The change in cross-section and curvature of the clavicle at the middle/lateral third junction of the clavicle seemingly makes it more susceptible to comminution following high-velocity injuries, presenting commonly with the vertically oriented ‘kickstand’ butterfly fragment. In our study, 27 patients (67.5%) presented with a fracture at the middle/lateral third junction of the clavicle and had a butterfly fragment or comminution, with the rest being midshaft clavicle fractures lateral to the midline. We noted the use of a lag screw at these fracture sites is difficult due to the flat surface of the clavicle. It is for this reason fiber wires were utilized to hold the fragments in reduction before plating. 

The variable sigmoid shape of the clavicle bone makes obtaining adequate fixation lateral to the fracture difficult when a regular superior locking plate is utilized [5,13]. Furthermore, it is challenging to achieve a three-screw bicortical fixation lateral to the fracture site, due to inadequate length and low screw density leading to decreased low pull-out strength. This shortcoming, however, is circumvented when using a Superior Clavicle LCP with Lateral extension, which has six screw holes in the lateral extension for increased screw density and pull-out strength at the lateral end of the clavicle. 

Limitations 

Our study demonstrates consistently satisfactory results in all patients managed with a Superior Clavicle LCP with Lateral extension. However, while beneficial it is retrospective and observational, with a small cohort and a relatively short period of follow-up (six months). 

We believe a large multicentre prospective randomized trial comparing Superior Clavicle LCP with Lateral extension to conventional fixation methods would prove invaluable in establishing evidence-based surgical management of Clavicle fractures [14]. Furthermore, a longer period of postoperative follow-up may provide better insights into post-op complications like implant-associated soft tissue irritation. A morphometric study of clavicle fracture patterns at the same time would also be beneficial.

## Conclusions

The Superior Clavicle LCP with Lateral extension appears to be ideally suited for the stabilization of displaced, comminuted diaphyseal clavicle fractures by restoring length and providing a stable construct with adequate screw purchases on either side of the fracture, especially for middle/lateral third fractures. Furthermore, patients reported positive postoperative outcomes, as demonstrated by VAS and QuickDASH scores on follow-up, with early mobilization and restoration of pre-injury mobility. The implant, based on our experience, is highly recommended for its excellent radiological and functional outcomes assisting in early return to pre-injury levels of activity.
